# The Effect of Cannabis on the Clinical and Cytokine Profiles in Patients with Multiple Sclerosis

**DOI:** 10.1155/2021/6611897

**Published:** 2021-02-05

**Authors:** Wessam Mustafa, Nadia Elgendy, Samer Salama, Mohamed Jawad, Khaled Eltoukhy

**Affiliations:** ^1^Faculty of Medicine, Mansoura University, Egypt; ^2^Faculty of Medicine, Delta University for Science and Technology, Egypt

## Abstract

**Background:**

Multiple studies have reported that cannabis administration in multiple sclerosis patients is associated with decreased symptom severity. This study was conducted to evaluate the prevalence of cannabis abuse in multiple sclerosis cases and to evaluate the effect of cannabis on serum cytokines in such cases. *Patients and Methods*. A total of 150 multiple sclerosis cases along with 150 healthy controls were included during the study period. All cases were subjected to history taking, neurological examination, and routine investigations. Cases were asked about cannabis intake which was confirmed by a urine test. Serum cytokines including IL-1, IL-2, IL-4, IL-10, IL-12, IL-17, IL-22, IFN-*γ*, IFN-*β*1, and TNF-*α* were ordered for all cases and controls.

**Results:**

Twenty-eight cases were cannabis abusers (MS/cannabis group, 18.67%). The remaining 122 cases represented the MS group. There was no significant difference between the three groups regarding age, disease duration, or MS type. Male gender was more predominant in the MS/cannabis group, and the number of relapses was significantly lower in the same group. Fifteen cases (53.6%) reported that their symptoms were improved by cannabis. Proinflammatory cytokines were significantly elevated in the MS group compared to the MS/cannabis and control groups. Additionally, anti-inflammatory cytokines had significantly lower values in the MS group compared to the MS/cannabis and control groups. Most clinical symptoms were significantly improved in the MS/cannabis group compared to the MS group apart from sexual dysfunction, bladder symptoms, and visual disturbances. Mild side effects of cannabis were also reported.

**Conclusion:**

Cannabis may have a positive impact on the cytokine and clinical profiles in cases with multiple sclerosis.

## 1. Introduction

Multiple sclerosis is an autoimmune disease of the central nervous system that is characterized by chronic inflammation, gliosis, demyelination, and neuronal loss. It is associated with multiple symptoms including pain, spasticity, fatigue, tremors, bladder problems, and heat intolerance. Such symptoms cause marked physical and cognitive disability for the affected patients [1].

The current treatment for multiple sclerosis includes two main categories: disease-modifying and symptomatic therapies. Disease-modifying drugs like immunomodulatory and/or immunosuppressive treatments are aimed at decreasing the number, severity, and duration of relapses. It also can help maintain remission and slow disease progression [2, 3]. Symptomatic therapies are prescribed to relieve the disabling symptoms of the disease. This group of drugs includes anticholinergics for bladder dysfunction, anticonvulsants for neuropathic pain, and botulinum toxins for spasticity [4]. Nevertheless, prescribing symptomatic treatment may be limited because of their potential toxicity [5].

Cases with multiple sclerosis usually seek nonconventional treatment options to control their symptoms. These modalities include complementary alternative medicine, herbal supplements, and cannabis [1, 6, 7].

Multiple reports have stated that cannabinoids extracted from cannabis including Delta9-Tetrahydrocannabinol (Delta9-THC), nabilone, and/or Delta9-THC hemisuccinate can control multiple sclerosis symptoms like spasticity, pain, and spasm [8, 9].

Studies conducted on animal models have reported that the activation of the cannabinoid (CB) 1 receptor could decrease neuropathic, visceral, and inflammatory pain [10, 11]. Furthermore, systemic administration of cannabinoid receptor ligands can provide analgesia in acute and chronic pain models [12]. CB2 receptors can have the same impact on neuropathic and inflammatory pain. It can also mediate antihyperalgesia in inflammatory pain states [13, 14]. In animals, the cannabinoids and the endocannabinoid system play a crucial role in decreasing muscle spasticity [15].

Only dronabinol and nabilone are the two cannabinoid derivatives that are approved by the US Food and Drug Administration (FDA). The former is used to manage anorexia in AIDS patients, whereas the latter is used for chemotherapy-induced nausea and vomiting [1]. Moreover, oral cannabis is approved by the American Academy of Neurology for managing pain and spasticity [7, 16].

Chong et al. have reported that 68% of multiple sclerosis cases reported improvement of their symptoms with cannabis administration [17]. In addition, Banwell et al. reported that the acceptance rate for the legalization of medical cannabis among Canadian patients was 54.3%. Also, the authors found that pain, anxiety, and spasticity were the most common reasons for marijuana use [18].

Since the 1970s, a significant increase in the number of drug abusers was noticed in Egypt [19]. The first Egyptian report regarding the prevalence of drug abuse reported that about 6.2% of the population were abusers [20], and this percentage continued to increase as reported by Hamdi and his coworkers [19].

This study had conducted at Mansoura University Hospitals. The aim was to evaluate the prevalence of cannabis abuse in multiple sclerosis cases, serum and gene expression of cytokines in cases versus controls. Also, to assess the effect of cannabis administration on cytokine levels in multiple sclerosis patients.

## 2. Patients and Methods

This is a prospective case-control study that was conducted at the Neurology Department, Mansoura University, during the period of one year (from January 2019 to January 2020).

A total of 150 cases diagnosed with multiple sclerosis, along with 150 healthy controls, were included in the current study. Cases were divided into two groups based on cannabis intake: MS group, which included cases who were not cannabis abusers, and MS/cannabis group, which included cannabis abusers. Only individuals aged more than 18 years were included,While subjects with other neurological diseases other than multiple sclerosis, substance abuse other than cannabis, psychiatric disorders, or whose responses regarding cannabis use were (don't know) and those who declined to answer were excluded.while subjects with other neurological diseases other than multiple sclerosis, with substance abuse other than cannabis, with psychiatric disorders, whose responses regarding cannabis use were “do not know,” and who declined to answer were excluded.

All cases were subjected to complete history taking, thorough neurological examination, and routine radiological investigations (brain MRI). The severity of symptoms was also evaluated by the Expanded Disability Status Scale (EDSS) [21]. In addition, assessment of the quality of life through the Multiple Sclerosis Quality of Life-54 (MSQOL-54, 52 items grouped in 12 subscales plus 2 single items) is the most used MS-specific health-related quality of life inventory [22]. All cases were asked if their symptoms were improved either with medical therapy alone or with medications together with cannabis.

All patients were subjected to cognitive assessment via the Mini-Mental State Exam (MMSE), and those who had cognitive impairment were excluded from the study.

Considering disease-modifying drugs (DMD), all patients were on first-line DMD that was interferon-beta, while those with secondary progressive disease were recruited before drug escalation to exclude drug effect in our study.

### 2.1. Exclusion Criteria

Exclusion criteria include the following: those who have an active form of the disease either symptoms suggestive of an active relapse or MRI findings suggestive of new T2 lesions in comparison to a previous MRI three months before recruitment or contrast-enhanced patches, as well as patients with cognitive decline and those who were on other forms of disease-modifying drugs other than interferon-beta or on DMD less than 1 year.

### 2.2. Cannabis Use Survey

All patients seen in the multiple sclerosis clinic at the Neurology Department, Mansoura University Hospital, during the study period were asked by the treating physician about cannabis use. A urine test was performed for MS cases who reported the use of cannabis as per standard clinical care. All cannabis users were asked to complete a modified nine-item survey on cannabis use to elaborate on their perception about cannabis use during the course of the disease [23].

Cases who used cannabis for at least the previous 3 months at least once weekly were included in the MS/cannabis group.

### 2.3. Analysis of Serum Cytokines

Blood samples were collected at 37°C by venipuncture from EDTA-anticoagulated blood samples. For subsequent analysis of cytokines, the collected serum was separated, aliquoted, and stored at −80°C. Enzyme-linked immunosorbent assay (ELISA) was used to measure the levels of the following cytokines according to the manufacturer's instructions (RayBiotech ELISA Kits, RayBiotech Life, Georgia, USA): proinflammatory cytokines (IL-1, IL-2, IL-6, IL-12, IL-17, IL-22, IFN-*γ*, and tumor necrosis factor-alpha) and anti-inflammatory cytokines (IL-4, IL-10, and IFN-*β*1).

Informed oral consent was obtained from all participants in the study. Moreover, the study was approved by the local ethical committee of Mansoura University.

### 2.4. Statistical Analysis

The collected data were coded, processed, and analyzed using the SPSS (Statistical Package for the Social Sciences) version 22 for Windows® (IBM SPSS Inc., Chicago, IL, USA). Data were tested for normal distribution using the Shapiro-Wilk test. Qualitative (categorical) data were represented as frequencies and relative percentages. The chi-squared test (*χ*^2^) was used to compare two or more independent groups of qualitative data. Quantitative data were expressed as mean ± SD (standard deviation) or median (range).

One-way analysis of the variance was used to compare three groups of independent parametric quantitative data, and the Kruskal-Wallis test was used to compare three groups of independent nonparametric quantitative data. Comparison between two independent groups of quantitative data was performed using the *t*-test for parametric data and the Mann-Whitney *U* test for nonparametric data. Significance test results were quoted as two-tailed probabilities, and *p* < 0.05 indicates a significant difference.

## 3. Results

There was no significant difference between the three study groups regarding age (*p* = 0.286). Regarding gender, females were predominant in the MS cases without cannabis (76.23%). On the other hand, males represented 71.4% of MS cannabis users and 52% of healthy controls. There was a significant difference between the three groups regarding that parameter (*p* < 0.001).

When it comes to the diseased cases, the age of diagnosis was not significantly different between the MS and MS/cannabis groups (*p* = 0.453). Likewise, disease duration had mean values of 13.78 and 12.17 years in both groups, respectively (*p* = 0.276). Surprisingly, the number of relapses was significantly lower in the MS/cannabis group (median = 2 vs. 1 in the MS group; *p* = 0.001). Moreover, the EDSS score was significantly improved in the same group compared to the MS group (*p* = 0.001).

Relapsing-remitting MS was the commonest form in both groups (75.4 vs. 75% in both groups, respectively), followed by the secondary progressive type (11.5 vs. 17.9% in both groups, respectively). Additionally, primary progressive MS was present in 9.02 and 7.1% of cases in both diseased groups, respectively. The progressive relapsing type was present in 4.1% of cases in the MS group while it was absent in the MS/cannabis group. The type of MS was not significant between the two groups (*p* = 0.486). The previous data are illustrated in Table 1.

In the MS/cannabis group, the duration of intake ranged between 2 and 14 years. Fifteen cases (53.6%) reported that their symptoms were improved by cannabis. Smoking was the commonest mode of cannabis intake, followed by vaping and mixed intake (14.3%). Dealers and family members were the two only sources for cannabis (53.6 and 46.4%, respectively).

Most of the cases in this group did not know the content of cannabis (92.2%). As regards the frequency of intake, 46.4 of cases were taking cannabis once daily, while 35.7% of cases took it twice per week. The remaining cases had cannabis either more than 1 time/day (10.7%) or once per week (7.1%).

Eight cases strongly agreed that cannabis improved their MS state (28.57%). Besides, 7 cases (25%) agreed with that. Conversely, 8 cases (28.6%) disagreed, while 3 cases (10.7%) strongly disagreed with the same concept.  Table 2 illustrates these data.

When it comes to the serum cytokine profile, it was evident that proinflammatory cytokines including IL-1, IL-2, IL-6, IL-17, IL-22, TNF, and IFN-*γ* were significantly elevated in the MS group compared to the MS/cannabis and control groups (*p* < 0.05). Additionally, anti-inflammatory cytokines had significantly lower values in the MS group compared to the MS/cannabis and control groups (*p* < 0.05). Generally speaking, although the MS/cannabis group had a worse cytokine profile compared to controls, it was significantly better compared to the MS group without cannabis intake. These data are illustrated in Table 3.

When MS cases were asked about symptom improvement with treatment using MSQOL-54, there was a significant improvement in the MS/cannabis group compared to the MS group regarding pain, spasm, fatigue, difficulty walking, anxiety, and mood changes (*p* < 0.05). On the contrary, the improvement of sexual dysfunction, bladder symptoms, and visual disturbances was not significant between the two groups (*p* > 0.05). These data are illustrated in Table 4.

Regarding cannabis drawbacks reported by our cases, sleepiness was the commonest encountered side effect (39.3%), followed by difficulty with concentration (35.7%) and hunger pain (28.6%). Other side effects included feeling hyper (21.4%), loss of balance (10.7%), paranoia (7.1%), nausea (7.1%), weakness (3.6%), and blurred vision (3.6%).  Table 5 illustrates these data.

## 4. Discussion

Multiple sclerosis is believed to be an autoimmune demyelinating CNS disease that is triggered by certain environmental or viral triggers on a susceptible genotype of the affected individual [24].

The clinical efficacy of cannabis in the management of multiple sclerosis symptoms remains a matter of debate. Cannabis appears to have a benefit in reducing pain in MS [17]. Nevertheless, another meta-analysis has denied the superiority of cannabis compared to minor opioids in the management of pain [25].

This study was conducted at Mansoura University Hospital and was aimed at evaluating the prevalence of cannabis abuse in multiple sclerosis cases and the effect of cannabis on serum cytokines and symptom improvement in such cases.

In our study, 28 out of 150 MS cases were cannabis abusers (18.67%). This is much lower than the reported percent in the literature. The etiology behind that could be explained by the fact that most MS cases are females, and cannabis abuse in Egypt is more common among males compared to females. The feeling of social disgrace may be the cause in some cases although patient confidentiality was ensured.

In a previous study, the overall prevalence of cannabis use was 39.9% in multiple sclerosis patients [1]. Previous studies reported the prevalence of 43% of multiple sclerosis cases in the United Kingdom [17] and Canada [26].

Patient general demographics did not differ between the study groups, apart from gender, which showed the significantly higher predominance of males. This is due to the fact that most of the cannabis abusers in Egypt are males [27]

Regarding clinical improvement, all studies symptoms showed significant improvement in the MS/cannabis cases compared to MS cases (*p* < 0.05), apart from sexual dysfunction, bladder symptoms, and visual disturbances. Of note, these last three symptoms also witnessed some degree of improvement, but it was not significantly different from the other group.

As multiple sclerosis cases usually have heterogeneous symptom profiles, it becomes more challenging while studying the effects of cannabinoids on this disease. Therefore, it may be hard to detect the effect on secondary outcomes when symptoms are not found in all study participants.

Likewise, in another study that included 37 multiple sclerosis cases, spasticity diminished by 2.74 points on the Ashworth scale, while pain decreased by 5.28 points on the visual analogue scale after cannabis smoking for 3 days. Nevertheless, cognition was significantly decreased as measured with the Paced Auditory Serial Addition Test [28]. This agrees with our findings regarding pain improvement.

Other researchers reported that 90% of the included MS cases reported improvement in both pain and spasticity [29]. Nevertheless, the other two studies reported no significant improvement regarding spasticity [5, 30].

Another study reported that following cannabis extract intake, the primary symptom score reduced from mean 74.36 to 48.89 and from 74.31 to 54.79 following the placebo (*p* > 0.05). Spasticity VAS scores were significantly reduced compared to the placebo (*p* = 0.001) [31].

Another study evaluated the safety, tolerability, and efficacy of two whole plant extracts of *Cannabis sativa* in multiple sclerosis patients with refractory lower urinary symptoms. After treatment, there was a significant decrease in urgency, frequency, number and volume of incontinence episodes, and nocturia. Moreover, pain, spasticity, and sleep disturbances were also improved. However, daily total voided, catheterized, and urinary incontinence pad weights also decreased significantly on both extracts [32].

Other trials have reported that cannabis could induce bladder function improvement [32], but not tremors [33].

In the current study, the number of relapses within the last five years was significantly lower in the MS/cannabis group (*p* = 0.001).

It was previously reported that endocannabinoid levels in the CNS might be increased during remission from an immune attack in patients with chronic neuroimmunological diseases. This in turn can limit symptom severity including spasticity and pain [15].

On the other hand, during the attack, there is a decrease in the endocannabinoid levels [34, 35], which may result in neuronal loss due to glutamate excitotoxicity and toxic accumulation of ions such as calcium [36, 37]. The decrease in endocannabinoid levels is attributed to the release of cytokines such as interferon-gamma by infiltrating T cells that disrupts the purinergic P2X7 receptor function which controls endocannabinoid responses by microglia [35]. This loss of endocannabinoid neuroprotection may contribute to nerve damage as a direct effect of the neuroimmune attack [38].

Based on the above findings, it is obvious that cannabis intake might prevent the incidence of relapse as it compensates for the relative deficiency essential for relapse to occur.

In the current study, fifteen cases (53.6%) reported that their symptoms were improved by cannabis. Eight cases strongly agreed that cannabis improved their MS state (28.57%). Besides, 7 cases (25%) agreed with that. Conversely, 8 cases (28.6%) disagreed, while 3 cases (10.7%) strongly disagreed with the same concept. Therefore, there was more tendency to accept cannabis as an effective modality in improving MS symptoms.

Recently, Cofield and his associates evaluated the interest in and use of marijuana before and after multiple sclerosis diagnosis in 5481 participants. The authors reported that 64% of the participants reported cannabis use before diagnosis, while 47% would consider cannabis as a possible supplementary treatment option for multiple sclerosis symptoms. In addition, 91% of respondents believed that cannabis administration should be legalized [39].

When it comes to the serum cytokine profile in our study, proinflammatory cytokines including IL-1, IL-2, IL-6, IL-17, IL-22, TNF, and IFN-*γ* were significantly elevated in the MS group compared to the MS/cannabis and control groups (*p* < 0.05). Additionally, anti-inflammatory cytokines including IL-4, IL-10, and IFN-*β*1 had significantly lower values in the MS group compared to the MS/cannabis and control groups (*p* < 0.05).

Multiple studies have reported that cannabinoids may slow progressive neurodegeneration [35, 40, 41]. Besides, experimental studies on multiple sclerosis showed that cannabinoids can inhibit the immune response in such cases [38]. These effects are mediated by both the CB1and CB2 receptors [42, 43].

The activation of cannabinoid receptors leads to the production of immunosuppressive molecules. Also, cannabis can control the release of neurotransmitters that lead to changes in the hormonal systems, including sex hormones, leptins, and glucocorticosteroids. These hormones can inhibit lymphocyte activation and cytokine production [44].

Furthermore, cannabinoids can inhibit T cell autoimmunity, antigen-presenting cell function, and production of proinflammatory cytokines such as interleukin-1 and tumor necrosis factor-alpha. It can also control the apoptosis of autoreactive cells [43, 45].

Sexton and his colleagues suggested that chronic cannabis use led to a global decrease of cytokines in both healthy controls and individuals with multiple sclerosis [46]. Conversely, other reports have noticed no changes regarding the levels of IFNc, IL-10, or IL-12 with cannabis administration [47, 48].

Other authors reported that in the presence of COR167 (high-affinity cannabinoid receptor agonist), there was a marked decrease or complete blocking of production of IL-2, IFN-gamma, TNF-alpha, IL-4, IL-5, IL-6, IL-17A, GCSF, and TGF-beta1. Conversely, a very high increase of secretion of IL-10, as well as a lower extent for IL-12, was observed [8]. Most of these findings agree with our results.

IL-17 is secreted mainly from T helper cells, and it plays a crucial role in CNS inflammation, especially in MS [49, 50]. Previous researchers stated that serum IL-17 levels were found to be significantly elevated in MS cases compared to healthy controls [51]. A previous study reported that neutralization of that cytokine resulted in significant attenuation of MS symptoms [52].

Regarding IL-22, it has been incriminated in blood-brain barrier breakdown and subsequent lymphocyte infiltration [53]. It can regulate immunity and contribute to MS severity [54]. Evidence from the existing literature suggests IL-22 dysregulation in MS cases [55] which may lead to astrocyte malfunction [56].

In addition, TNF-*α* was reported to be significantly overexpressed in MS cases [57]. It is a potent inflammatory cytokine which is secreted from different immunity cells including macrophages, monocytes, and differentiated T cells [58]. However, another study reported that TNF-*α* may have a dual function in the CNS, as its deficiency led to more inflammation and demyelination in animals [59]. Based on these heterogeneous data, it is recommended to conduct more studies regarding that cytokine to precisely define its role in CNS diseases.

IFN-*γ*, which is secreted from T cells and natural killer cells, has an important role in the autoimmune pathogenesis of MS. An increase in its levels was detected during symptomatic disease, while it decreased significantly during remission [60, 61]. Moreover, IFN-*γ* antibody treatment delayed disease progression in a previous study [62].

An IL-1 increase was detected in cases with systemic inflammation and brain damage, along with infections [63]. It induces polarization of T cells toward Th17 cells, which are the key cells in MS [64]. However, other studies showed that IL-1 has a neuroprotective effect mediated by the nerve growth factor [65].

As MS is mainly an autoimmune disease, its role in Th1 cell expansion is thought to share in the pathogenesis of MS. Besides, its levels were also correlated with disease activity [66].

Based on the previous data, a decrease in the levels of the previously mentioned proinflammatory cytokines surely will help improve patient condition and relieve distressing symptoms as experienced in the current study.

On the other hand, IL-4 causes a decrease in the proinflammatory cytokines including IL-1, IL-6, IL-8, and TNF-*α* [67]. Furthermore, it can inhibit macrophage activity [68]. Animals deficient in IL-4 showed an increased proinflammatory response and axonal injury [69].

Similarly, IL-10 can inhibit the release of proinflammatory cytokines [70]. It was found to decrease the severity of autoimmune diseases in experimental animals through its interaction with IL-21 [71].

IFN-*β* has shown significant treatment effects in relapsing MS patients by reducing physical disability, exacerbation frequency, and disease activity [72]. It mediates its effects through increasing IL-10 expression, modulating IgG production, and decreasing proinflammatory cytokines [73].

The decrease in the previously mentioned three anti-inflammatory cytokines will have a positive impact on disease progression, as illustrated in our study.

As regards cannabis drawbacks encountered in the current study, sleepiness was the commonest encountered side effect (39.3%), followed by difficulty with concentration (35.7%) and hunger pain (28.6%). Other side effects included feeling hyper (21.4%), loss of balance (10.7%), paranoia (7.1%), nausea (7.1%), weakness (3.6%), and blurred vision (3.6%).

Despite symptom improvement, some side effects must be taken into consideration, especially in multiple sclerosis cases [74]. The potential for cognitive dysfunction is still a concern in cases with preexisting dysfunction. Other physicians expressed their caution regarding cannabis use in the elderly and persons with psychosis [75].

The most common reported side effects include dizziness, euphoria, difficulty with concentration, diarrhea, and dry mouth. In most reviews, these symptoms were described to be mild to moderate in severity [76].

Another study reported that the most common of these side effects were feeling “quiet and mellow” (41%), sedated (35%), euphoric (“giggly,” 27%), “lazy” (23%), and always feel hungry (increased appetite, 29%). Three subjects reported paranoia, another three experienced exaggerated weakness, and one case reported hallucinations [17].

Wade and his coworkers reported that there were no significant adverse effects on cognition or mood and intoxication was generally mild [31].

Our study has some advantages; it discussed the most relevant cytokines involved in the MS pathway. Also, the clinical response was also evaluated as well. We should have evaluated these cytokines in CSF, but it was difficult to convince 150 healthy controls to have a lumbar puncture.

On the contrary, some limitations of the current study are also present. First, it is a single-center study. In addition, cannabis use by patients was for creational not therapeutic purposes. The dose sufficient to improve symptoms was also unknown as all cases did not know the exact dose taken for sure. It is recommended to conduct more studies including more cases from different centers. Also, medical cannabis should be commenced for such cases to determine the effective dose for symptom improvement and to be sure of the different ingredients of the administered drug, as the majority of cannabis in Egypt is of different purity.

## 5. Conclusion

Based on our results, cannabis may have a positive impact on the cytokine and clinical profiles in cases with multiple sclerosis. However, more studies should be conducted regarding that perspective to reach a final decisive result.

## Figures and Tables

**Table 1:** Demographic data and clinical criteria in the three study groups.

	MS cases (*N* = 122)	MS/cannabis (*N* = 28)	Control (*N* = 150)	*p* value	
Age (years)	38.6 ± 4.19	38.4 ± 4.52	38.75 ± 5.01	0.286^a^	
Sex					
Males	29 (23.77%)	20 (71.4%)	78 (52%)	<0.001^b^	<0.001^c^
Females	93 (76.23%)	8 (28.6%)	72 (48%)		<0.001^d^
					0.005^e^
Age at diagnosis of MS (years)	25.34 ± 2.18	26.63 ± 2.42			0.453^c^
Disease duration (years)	13.78 ± 3.44	12.17 ± 2.71			0.276^c^
Number of relapse	2 (1-5)	1 (0-2)			0.001^c^
EDSS	4 (3–4.5)	1.5 (1–2)			0.001^c^
Types of MS					
RRMS	92 (75.4%)	21 (75%)			0.486^c^
SPMS	14 (11.5%)	5 (17.9%)			
PPMS	11 (9.02%)	2 (7.1%)			
PRMS	5 (4.1%)	0 (0%)			

PPMS: primary progressive multiple sclerosis; PRMS: progressive relapsing multiple sclerosis; RRMS: relapsing-remitting multiple sclerosis; SPMS: secondary progressive multiple sclerosis. ^a^One-way ANOVA test/Kruskal-Wallis test. ^b^Comparison between the three groups using the chi-squared test. ^c^Comparison between MS cases and MS cases (chronic cannabis users). ^d^Comparison between MS cases and controls. ^e^Comparison between MS cases (chronic cannabis users) and controls.

**Table 2:** Summary of cannabis use survey responses (*N* = 28).

Questions	Answers options	Answers	
		*N*	Percent
Sex	Males	20	71.4%
	Females	8	28.6%
Duration of intake	Years	6 (2-14)	
Do you use cannabis to treat your MS?	Yes	15	53.6%
	No	13	46.4%
Method of use of cannabis	Smoking	14	50%
	Goza or vaping	4	14.3%
	Eating	2	7.1%
	Bong/water pipes	1	3.6%
	Drinks (tea/coffee)	3	10.7%
	Mixed	4	14.3%
Where do you get cannabis from?	Homegrown	0	0%
	Family members/friends/colleagues	13	46.4%
	Dealers	15	53.6%
	Medical prescription	0	0%
Is it easier or harder to get cannabis in the last few years?	Easier	10	35.71%
	Harder	5	17.86
	Same as before	13	46.43
Times of intake	Once/week	2	7.1%
	2 times or more/week	10	35.7%
	Once daily	13	46.4%
	More than 1 time/day	3	10.7%
How many milligrams taken every time?	I do not know	28	100%
	I know (measurement)		
Do you know the contents of your cannabis?	Yes	2	7.1%
	No	26	92.9%
Cannabis improve my MS	Strongly agree	8	28.57%
	Agree	7	25%
	Neither agree or disagree	2	7.14%
	Disagree	8	28.6%
	Strongly disagree	3	10.7%

**Table 3:** Analysis of serum cytokines in the study groups.

	MS cases (*N* = 122)	MS/cannabis (*N* = 28)	Control (*N* = 150)	*p* value	
IL-1 (pg/ml)	27.6 ± 5.83	14.16 ± 3.26	4.29 ± 1.13	<0.001^a^	<0.001^c^
					<0.001^d^
					0.001^e^
IL-2 (pg/ml)	29.77 ± 6.19	17.43 ± 4.98	5.32 ± 2.28	<0.001^a^	<0.001^c^
					<0.001^d^
					<0.001^e^
IL-4 (pg/ml)	10.2 ± 3.28	13.29 ± 3.79	16.88 ± 4.15	0.021^a^	0.128^c^
					0.005^d^
					0.075^e^
IL-6 (pg/ml)	44.85 ± 9.22	21.15 ± 5.38	9.35 ± 3.29	<0.001^a^	<0.001^c^
					<0.001^d^
					<0.001^e^
IL-10 (pg/ml)	15.44 ± 3.87	27.11 ± 6.49	36.59 ± 7.14	<0.001^a^	<0.001^c^
					<0.001^d^
					<0.001^e^
IL-12 (pg/ml)	96.15 ± 19.37	53.88 ± 13.23	40.47 ± 10.70	<0.001^a^	<0.001^c^
					<0.001^d^
					<0.001^e^
IL-17 (pg/ml)	23.74 ± 5.33	12.82 ± 3.64	3.11 ± 0.75	<0.001^a^	<0.001^c^
					<0.001^d^
					<0.001^e^
IL-22 (pg/ml)	30.45 ± 7.58	22.18 ± 5.87	6.17 ± 2.38	<0.001^a^	<0.001^c^
					<0.001^d^
					<0.001^e^
TNF (pg/ml)	43.63 ± 10.58	25.05 ± 7.49	12.13 ± 3.97	<0.001^a^	<0.001^c^
					<0.001^d^
					<0.001^e^
IFN-*β*1 (pg/ml)	11.42 ± 3.77	13.27 ± 3.25	14.07 ± 4.19	0.094^a^	0.238^c^
					0.054^d^
					0.126^e^
IFN-*γ* (pg/ml)	19.27 ± 5.33	15.24 ± 4.74	8.55 ± 3.19	0.005^a^	0.048^c^
					<0.001^d^
					0.001^e^

IL: interleukin; IFN: interferon; TNF: tumor necrosis factor. ^a^One-way ANOVA test/Kruskal-Wallis test. ^c^Comparison between MS cases and MS cases (chronic cannabis users). ^d^Comparison between MS cases and controls. ^e^Comparison between MS cases (chronic cannabis users) and controls.

**Table 4:** Analysis of symptom improvement in cases with MS.

	MS cases (*N* = 122)	MS/cannabis (*N* = 28)	*p* value
Pain	62 (50.8%)	19 (67.9%)	0.019^b^
Limb spasm	73 (59.8%)	22 (78.6%)	0.011^b^
Fatigue	29 (23.77%)	10 (35.7%)	0.045^b^
Walking difficulties	69 (56.6%)	18 (64.3%)	0.043^b^
Sexual dysfunction	64 (52.5%)	14 (50%)	0.638^b^
Anxiety	53 (43.4%)	18 (64.3%)	0.001^b^
Altered mood	49 (40.2%)	7 (25%)	0.025^b^
Visual disturbances	58 (47.5%)	13 (46.4%)	0.876^b^
Bladder symptoms	60 (49.18%)	14 (50%)	0.956^b^

^b^Chi-squared test.

**Table 5:** Side effects in the MS cases (chronic cannabis users).

	MS/cannabis (*N* = 28)
Sleepiness	11 (39.3%)
Hunger pain	8 (28.6%)
Feeling hyper and chatty	6 (21.4%)
Loss of balance	3 (10.7%)
Difficulty with concentration	10 (35.7%)
Weakness	1 (3.6%)
Blurred vision	1 (3.6%)
Paranoia	2 (7.1%)
Hallucinations	0 (0%)
Nausea	2 (7.1%)

## Data Availability

The datasets generated and/or analyzed during the current study are not publicly available due to current Mansoura University regulations & Egyptian legislation but are available from the corresponding author on reasonable request and after institutional approval.
